# Advance Without Cut and Retrograde Removal of Embedded Fishhook; Introducing a Novel Technique

**DOI:** 10.22037/aaem.v10i1.1403

**Published:** 2022-01-01

**Authors:** AbdolGhader Pakniyat, Kourosh Akhbari, Fatemeh Radfar

**Affiliations:** 1 Department of Emergency Medicine, Kosar Hospital, Kurdistan University of Medical Sciences, Sanandaj, Iran.; 2 Men's Health and Reproductive Health Research Center, Shahid Beheshti University of Medical Sciences, Tehran, Iran.; 3 Emergency Medicine Department, Shahid Beheshti University of Medical Sciences, Tehran, Iran.

**Keywords:** Foreign Bodies, Wounds and Injuries, soft tissue injuries, Emergency Service, Hospital

## Abstract

Removing embedded fishhook without causing further tissue damage from the barbed nature of the hook is a challenge in emergency department (ED). The four most commonly used techniques include advance and cut, string-yank, needle cover, and retrograde removal. This study aims to describe a modified push- through technique without cutting the barb, namely advance without cut and retrograde removal, as an effective technique of successful removal of fishhooks. There is no risk of additional injury to patients and healthcare staff, and the technique does not need tools that are not generally readily available in EDs.

**Dear Editor;**

Removing embedded fishhook without causing further tissue damage from the barbed nature of the hook is a challenge in emergency department (ED). The four most commonly used techniques include advance and cut, string-yank, needle cover, and retrograde removal (1, 2). Most Fishhooks are embedded in the skin and cutaneous soft tissue of hand, foot, and face. There are no absolute contraindications to fishhook removal. While the external injury often seems minimal, internal injuries can be dangerous, particularly when the barbed hook is lodged near a blood vessel, tendon, or nerve. Surgical consultation may be obtained for appropriate removal and repair (3). The choice of removal technique is different and depends on anatomical location of the affected body part, the depth of penetration, and type and size of fishhook. X- rays may aid in determining the type of fishhook and depth of penetration in difficult cases (4-6).

This study presents 2 patients; a 43-year-old male and a 24-year-old male with superficially embedded fishhook presenting to ED of Kosar Hospital, Sanandaj, Iran during August 2021. The fishhooks were embedded in dorsal of right hand (case 1) and first and second volar of distal phalanx of left hand (case 2) with no significant hemorrhage. All sensory and motor examinations were normal. The risks and benefits of the procedure was explained to the patients initially and then push-through technique was selected for removal of fishhooks. After the skin and hook were prepped with betadine solution, in order to reduce the pain, skin overlying the point of the hook was anesthetized, either by local infiltration with 1 percent lidocaine or by digital block. After anesthesia was obtained, the shank of the hook was grasped with a hemostat and then it was advanced into the wound until the barbed end protruded through the skin. Since a clean wire cutter was not available, using a needle holder, the barb was clamped and bent over body of the bend part. When the fishhooks were transformed to barbless hook, they were back out easily with no additional injury (figure 1). 

After washing with normal saline and dressing with antibiotic ointment, the patients were discharged with recommendations to hold the wound in warm water two to three times per day until healing is established and advised them to return if any signs of infection appear. There was no complication in the follow-up (10 days later). 

The advance and cut causes minimal additional soft tissue trauma and is most effective. In this technique, after the advancement of fishhook into the wound until the barb reemerges from the skin, the barb is cut off with a wire cutter and the remainder of the hook is then backed out of the wound. Cutting the barb requires taking precautions to avoid inadvertent injury from the barb, so the patient, clinician, and other care providers in the room should wear protective eye gear (1, 2, 7, 8)

In advance without cut and retrograde method, removal of the fishhook is done using medical devices like needle holder or hemostat and we do not need any tools that are not available in EDs, like wire cutter. Before retrograding the hook, it is recommended to make sure that the barb is bent and the fishhook is made completely barbless. The procedure must be stopped if the physician feels significant resistance when pulling the hook, which may be due to incomplete bending of the barb, as additional tissue injury and pain can be presumed. 

**Figure 1:**
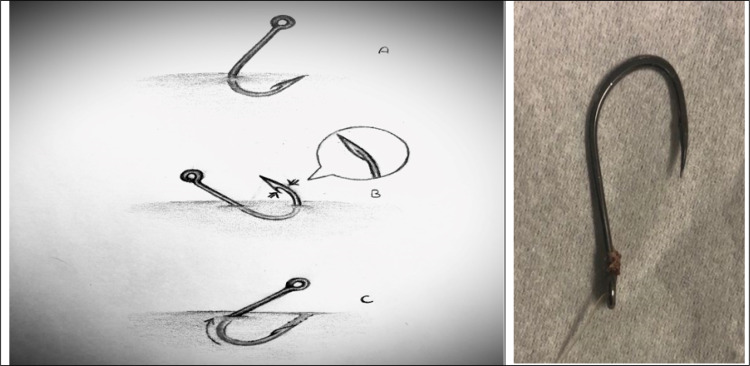
A: the shank of the hook is grasped with a hemostat and then it is advanced into the wound until the barbed end protrudes through the skin; B: using a needle holder the barb is clamped and bent over the body of the bend part (made barbless); C: the hook is then removed in a retrograde manner

## Conclusion:

Advance without cut and retrograde method can be considered as one of the effective and safe techniques. It is easy to do and all physicians in EDs can do the procedure after some training. 

## 1. Declarations

### 1.1 Acknowledgements

We would like to thank Dr. Sepideh Moradkhani for her support during the initial write up and drawing the picture of this paper.

### 1.2 Authors’ Contribution

Study concept and design of the manuscript: A.P; Drafting, analysis, and interpretation of data: A.P, K.A and F.R; critical revision of the manuscript for important intellectual content: A.P, K.A and F.R; study supervision: A.P.

### 1.3 Financial Disclosure

We had no financial interests related to the materials in the manuscript.

### 1.4 Funding/Support

None declared.
